# Prevalence, Demographic Correlates, and Perceived Impacts of Mobile Health App Use Amongst Chinese Adults: Cross-Sectional Survey Study

**DOI:** 10.2196/mhealth.9002

**Published:** 2018-04-26

**Authors:** Zhenzhen Xie, Ahmet Nacioglu, Calvin Or

**Affiliations:** ^1^ Department of Industrial and Manufacturing Systems Engineering The University of Hong Kong Hong Kong China (Hong Kong)

**Keywords:** mHealth, mobile health apps, prevalence, demographic correlates, health behavior

## Abstract

**Background:**

Mobile health apps have changed the way people obtain health information and services and advance their understanding and management of their health. Although many health apps are available, little is known about the prevalence of their use for different purposes, whether such use is associated with demographic characteristics, and the impacts of their use on health knowledge and management.

**Objective:**

The main objectives of this study were to examine the prevalence, extent, and demographic correlates of health app use and the perceived impacts of health app use on increased health knowledge and improved health condition management.

**Methods:**

We conducted a cross-sectional questionnaire survey of 633 Chinese adults randomly drawn from the general population in Hong Kong.

**Results:**

Of the 633 participants, 612 (96.7%) reported using mobile devices. Of them, 235 (38.4%) reported using multiple types of health apps. The most-used type of health app was about healthy living information (197/612, 32.2%), followed by measuring/recording vital signs (80/612, 13.1%), health and medical reminders (64/612, 10.5%), recovery and rehabilitation information (42/612, 6.9%), diagnosis assistance (28/612, 4.6%), emergency services (16/612, 2.6%), telehealth (11/612, 1.8%), and “other” (19/612, 3.1%). Multivariate logistic regression analysis found that health app users were more likely to be women (odds ratio [OR] 1.68, 95% CI 1.14-2.48, *P*=.01) of a higher self-rated social class (OR 3.66, 95% CI 1.11-12.11, *P*=.03). Participants who worked in education/culture/academia (OR 2.31, 95% CI 1.16-4.59, *P*=.02) or disciplinary forces (OR 5.07, 95% CI 1.25-20.62, *P*=.02) were more likely to believe that using health apps could increase their health knowledge; participants working in education/culture/academia were also more likely to believe that using health apps could improve the effectiveness of health condition management (OR 2.18, 95% CI 1.10-4.34, *P*=.03).

**Conclusions:**

Effort should be made to promote health app use, especially to demographic groups that are currently less likely to use health apps (eg, males, individuals from lower social classes). From the public health perspective, guidelines could be developed to help individuals identify quality health apps that meet their needs. Moreover, app developers could improve the usability of health apps to promote health app use.

## Introduction

The rapid development of mobile devices, particularly mobile phones, and internet technology has led to a surge of interest in using mobile apps to implement mHealth [1-3]. There are more than 325,000 health apps available, covering various health topics such as disease management, healthy lifestyles, self-diagnosis, and emergency services [4-6]. The number of health app downloads is high, with more than 3 billion in 2015, and it is growing at a rate of more than 7% each year [4].

Several studies have examined the prevalence of health app use and the association between demographics and health app use. For instance, Krebs and Duncan [7] found that 58.2% of US mobile phone users had downloaded a health app and that health app users tended to be younger, were of Latino/Hispanic ethnicity, had higher income, were more educated, and had higher body mass index. Based on a US survey, Carroll et al [8] found that health app users tended to be younger, female, more educated, high income earners, and individuals in excellent health. Ernsting et al’s [9] German survey results revealed that 20.5% of mobile phone users used health apps and that health app use was related to age, first language, internet use, chronic conditions, health behaviors, and health literacy. The demographics associated with health app use can vary greatly across different app types (eg, healthy living information, diagnosis assistance, health and medical reminders), but previous studies have focused on general health app use overall and have not examined the demographic correlates of each type of health app separately.

Moreover, although health apps aim to offer their users health benefits, such as increased health knowledge and improved health management [10-13], relatively little is known about whether users find or perceive that health apps confer such benefits. Although studies have examined the perceived impacts of using health apps, they focused on only one or two types of health apps for a specific population (eg, young adults, sports dietitians) [14,15]. The demographic correlates of the perceived impacts of health app use have also been little studied.

In this study, we set out to first explore the prevalence and extent of mobile device use and mobile internet access. We then examined the prevalence, extent, and demographic correlates of health app use in general and by app type, along with individuals’ perceived impacts of health app use on increased health knowledge and improved health condition management, and the demographic correlates of these perceptions.

## Methods

### Design

The study used a cross-sectional questionnaire survey design. Multimedia Appendix 1 presents the questionnaire we used. The survey collected demographic data (age, gender, self-rated social class, education, and occupation), mobile device use, mobile internet access, health app use, and perceived impacts of health app use on increased health knowledge and improved health condition management. No compensation was given for participation in the study. The study was approved by the ethics committee of the University of Hong Kong, and informed consent was obtained from all participants.

### Participants

The sample consisted of 633 adults who were selected using convenience sampling and stratified by age group (18-29, 30-44, 45-59, and ≥60 years) and gender. Individuals who met the following criteria were eligible for the study: aged 18 years or older, able to understand written and spoken Chinese, and able to understand the questionnaire.

### Procedure

Research assistants randomly approached individuals in public areas (eg, shopping malls, subway stations, residential neighborhoods, and parks), introduced the study to them, asked them if they would be willing to participate in the study, and confirmed their eligibility. Those who agreed and were eligible were asked to complete the questionnaire. The research assistants read the questions aloud and recorded the responses for individuals who asked them to administer the questionnaire. The data were collected between April 2016 and March 2017.

### Data Analysis

Two research assistants independently entered the data and crosschecked them for accuracy. Some participants did not respond to some questions. Responses that contained errors, such as reporting desktop computer use as mobile device use or social media app use as health app use, were excluded from the data analysis. Participants’ self-ratings of social class, which were obtained on a 9-point scale anchored at the extremes by poor (1) and rich (9) were collapsed into three categories: lower (1-3), middle (4-6), and upper (7-9). Descriptive statistics were computed for demographic variables, mobile device use, mobile internet access, health app use, and perceived impacts of health app use. Multivariate logistic regressions were conducted to assess the demographic correlates of health app use and perceived impacts of health app use. The odds ratios and 95% confidence intervals were calculated. All the analyses were performed using STATA 14.

## Results

### Sample Characteristics

Table 1 shows the demographic characteristics of the sample (N=633). The mean age of the sample was 45.25 years (SD 17.44). Of all the participants, only 2.2% (14/633) reported that they were from an upper social class, with 63.5% (402/633) from a middle social class, and 33.2% (210/633) from a lower social class.

### Prevalence and Extent of Mobile Device Use and Mobile Internet Access

The prevalence and extent of the participants’ mobile device usage are presented in Table 2. Overall, 96.7% (612/633) of the participants reported using mobile devices, and 90.5% (573/633) reported using mobile phones. Of the mobile device users, 90.8% (556/612) reported having internet access on their devices, and only 7.2% (44/612) reported not having mobile internet access (2% did not respond to this question).

**Table 1:** Demographic characteristics of the sample (N=633).

Characteristic		n (%)
**Gender**		
	Male	325 (51.3)
	Female	307 (48.5)
	No response	1 (0.2)
**Age (years)**		
	18-29	156 (24.6)
	30-44	158 (25.0)
	45-59	156 (24.6)
	≥60	158 (25.0)
	No response or erroneous data	5 (0.8)
**Self-rated social class**		
	Lower	210 (33.2)
	Middle	402 (63.5)
	Upper	14 (2.2)
	No response	7 (1.1)
**Education level**		
	No schooling completed	8 (1.3)
	Some primary school	18 (2.8)
	Completed primary school	37 (5.8)
	Some secondary school	54 (8.5)
	Completed secondary school	175 (27.7)
	Diploma, advanced diploma, associate degree or equivalent	90 (14.2)
	Bachelor’s degree	154 (24.3)
	Master’s degree	77 (12.2)
	Doctoral degree	19 (3.0)
	Other	1 (0.2)
**Occupation**		
	Service	83 (13.1)
	Sales	24 (3.8)
	Catering	13 (2.1)
	Finance	41 (6.5)
	Engineering	49 (7.7)
	Art	4 (0.6)
	Education/culture/academia	64 (10.1)
	Administration/professional	35 (5.5)
	Office/white-collar worker	35 (5.5)
	Disciplinary forces	7 (1.1)
	Student	65 (10.3)
	Housewife/househusband	45 (7.1)
	Unemployed/awaiting job assignment	16 (2.5)
	Retiree	122 (19.3)
	Other	24 (3.8)
	No response	6 (1.0)

### Prevalence, Extent, and Demographic Correlates of Health App Use

Table 3 presents the prevalence and demographic correlates of health app use in general. Overall, 38.4% (235/612) of the mobile device users reported using health apps, and 60.3% (369/612) reported not using any health apps (1.3% did not respond to this question).

The logistic regression results showed that females (odds ratio [OR] 1.68, 95% CI 1.14-2.48, *P*=.01) and participants in higher self-rated social classes (middle: OR 1.43, 95% CI 0.94-2.16, *P*=.09; upper: OR 3.66, 95% CI 1.11-12.11, *P*=.03) were more likely to use health apps.

Multimedia Appendix 2 presents the prevalence, extent (the mean length of time spent on each occasion of use, in minutes), and demographic correlates of health app use by type. The most prevalent health app type was healthy living information, which 32.2% (197/612) of the mobile device users reported using, followed by measuring/recording vital signs (80/612, 13.1%), health and medical reminders (10.5%, 64/612), recovery and rehabilitation information (6.9%, 42/612), diagnosis assistance (28/612, 4.6%), emergency services (16/612, 2.6%), telehealth (11/612, 1.8%), and “other” (19/612, 3.1%).

Users of health and medical reminder apps were more likely to be female (OR 2.44, 95% CI 1.31-4.52, *P*=.01) and less likely to be housewives/househusbands (OR 0.16, 95% CI 0.03-0.82, *P*=.03) or retirees (OR 0.23, 95% CI 0.06-0.88, *P*=.03). Participants who had retired were also less likely to use diagnosis assistance apps (OR 0.06, 95% CI 0.01-0.43, *P*=.01). Participants who had completed secondary school (OR 35.68, 95% CI 2.85-447.02, *P*=.01), or a diploma, advanced diploma, associate degree or equivalent (OR 15.55, 95% CI 1.07-225.77, *P*=.04) were more likely to use diagnosis assistance apps. Participants in higher self-rated social classes (middle: OR 2.74, 95% CI 0.88-8.51, *P*=.08; upper: OR 111.09, 95% CI 4.31-2828.89, *P*=.004) were also more likely to use diagnosis assistance apps. In addition, participants in higher self-rated social classes were more likely to use apps for healthy living information (middle class: OR 1.55, 95% CI 1.01-2.40, *P*=.046; upper class: OR 2.84, 95% CI 0.88-9.08, *P*=.08), recovery and rehabilitation information (middle class: OR 2.98, 95% CI 1.14-7.80, *P*=.03; upper class: OR 15.01, 95% CI 2.03-110.78, *P*=.01), and measuring/recording vital signs (middle class: OR 2.31, 95% CI 1.16-4.60, *P*=.02; upper class: OR 8.32, 95% CI 2.16-32.05, *P*=.002).

### Perceived Impacts of Health App Use on Increased Health Knowledge and Improved Health Condition Management

**Figure 1:**
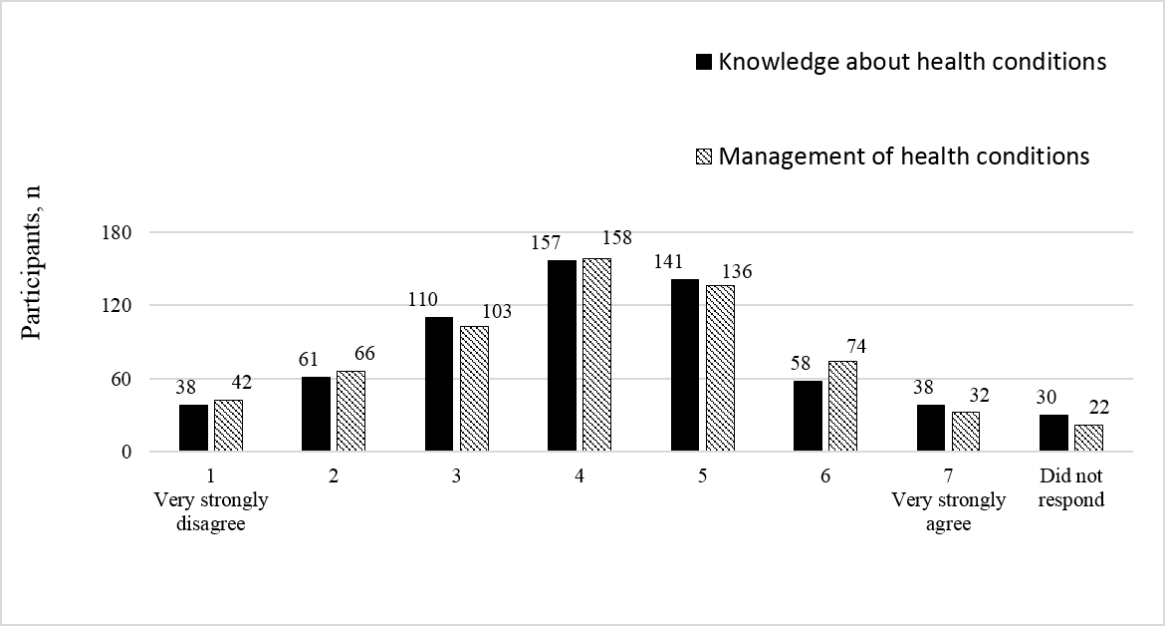
Frequency distribution of perceived impacts of health app use.

Figure 1 shows the frequency distribution of the participants’ perceived impacts of health app use on increased knowledge about health conditions and improved health condition management. The participants rated their agreement with the statement “using mobile health apps can increase your knowledge about and improve the effectiveness of the management of your health conditions” on a 7-point scale, with 1=very strongly disagree and 7=very strongly agree. For increase in health condition knowledge, 37.4% (237/633) of the participants gave a rating of 5 or above, and 33% (209/633) gave a rating of 3 or below. For health management improvement, 38.2% (242/633) of the participants gave a rating of 5 or above and 33.3% (211/633) gave a rating of 3 or below.

Table 4 presents the means and standard deviations of the perceived impacts of health app use on increased health knowledge and improved health condition management by demographic characteristics and demographic correlates of perceptions. The analysis showed that participants working in education/culture/academia (OR 2.31, 95% CI 1.16-4.59, *P*=.02) and disciplinary forces (OR 5.07, 95% CI 1.25-20.62, *P*=.02) tended to believe that using health apps could increase their health knowledge. In addition, participants working in education/culture/academia (OR 2.18, 95% CI 1.10-4.34, *P*=.03) and those who reported “other” occupations (eg, health care, sports, media, social work; OR 2.50, 95% CI 1.07-5.82, *P*=.03) were more likely than other types of workers to believe that using health care apps could improve the effectiveness of their health condition management.

**Table 2:** Prevalence and extent of mobile device use (N=633).

Mobile device	n (%)	Hours spent using the device daily, mean (SD)
Mobile phone	573 (90.5)	4.0 (3.6)
Feature phone	49 (7.7)	1.5 (2.2)
Tablet computer	209 (33.0)	2.5 (2.5)
Other	23 (3.6)	4.5 (3.6)
Not using any mobile devices	18 (2.8)	—
No response or erroneous data	3 (0.5)	—

**Table 3:** Prevalence and demographic correlates of use of any type of health app (N=612). OR: odds ratio.

Demographic characteristics		n (%)	OR (95% CI)	*P*
Total		235 (38.4)		
**Gender**				
	Male	108 (33.2)	1	
	Female	126 (41)	1.68 (1.14-2.48)	.01
**Age (years)**				
	18-29	61 (39.1)	1	
	30-44	70 (44.3)	1.06 (0.61-1.81)	.84
	45-59	59 (37.8)	0.96 (0.53-1.74)	.90
	≥60	41 (25.9)	0.69 (0.31-1.51)	.35
**Self-rated social class**				
	Lower	66 (31.4)	1	
	Middle	156 (38.8)	1.43 (0.94-2.16)	.09
	Upper	9 (64.3)	3.66 (1.11-12.11)	.03
**Education level**				
	No schooling completed	0 (0)	1	
	Some primary school	3 (16.7)	0.26 (0.05-1.43)	.12
	Completed primary school	10 (27.0)	0.40 (0.10-1.58)	.19
	Some secondary school	22 (40.7)	0.66 (0.19-2.28)	.51
	Completed secondary school	55 (31.4)	0.39 (0.13-1.20)	.10
	Diploma, advanced diploma, associate degree or equivalent	31 (34.4)	0.46 (0.15-1.42)	.18
	Bachelor’s degree	67 (43.5)	0.50 (0.17-1.44)	.20
	Master’s degree	37 (48.1)	0.65 (0.22-1.92)	.44
	Doctoral degree	10 (52.6)	1	
	Other	0 (0)	1	
**Occupation**				
	Service	36 (43.4)	1	
	Sales	9 (37.5)	0.70 (0.26-1.87)	.47
	Catering	1 (7.7)	0.14 (0.02-1.17)	.07
	Finance	19 (46.3)	0.87 (0.37-2.06)	.75
	Engineering	17 (34.7)	0.59 (0.26-1.35)	.21
	Art	2 (50.0)	1.01 (0.12-8.22)	>.99
	Education/culture/academia	26 (40.6)	0.55 (0.25-1.22)	.14
	Administration/professional	21 (60.0)	1.48 (0.60-3.64)	.40
	Office/white-collar worker	15 (42.9)	0.77 (0.31-1.94)	.59
	Disciplinary forces	3 (42.9)	0.80 (0.16-4.03)	.79
	Student	22 (33.8)	0.46 (0.20-1.07)	.07
	Housewife/househusband	14 (31.1)	0.50 (0.22-1.17)	.11
	Unemployed/awaiting job assignment	5 (31.3)	0.60 (0.18-1.97)	.40
	Retiree	32 (26.2)	0.63 (0.29-1.40)	.26
	Other	11 (45.8)	1.09 (0.40-2.96)	.86

**Table 4:** Means and standard deviations of perceived impacts of health app use on increased health knowledge and improved health condition management, and demographic correlates (N=633). N/A: not applicable; OR: odds ratio.

Demographic characteristics		Health knowledge			Health condition management		
		Mean (SD)	OR (95% CI)	*P*	Mean (SD)	OR (95% CI)	*P*
**Gender**							
	Male	4.11 (1.51)	1		4.03 (1.55)	1	
	Female	3.97 (1.55)	0.81 (0.59-1.13)	.22	4.02 (1.54)	1.08 (0.78-1.49)	.64
**Age (years)**							
	18-29	3.93 (1.29)	1		3.91 (1.45)	1	
	30-44	4.07 (1.48)	1.07 (0.67-1.70)	.79	4.12 (1.40)	1.16 (0.73-1.84)	.53
	45-59	4.03 (1.55)	1.09 (0.66-1.80)	.74	4.10 (1.57)	1.33 (0.80-2.20)	.27
	≥60	4.15 (1.76)	1.51 (0.77-2.97)	.23	3.99 (1.75)	1.34 (0.69-2.60)	.39
**Self-rated social class**							
	Lower	3.89 (1.56)	1		3.80 (1.57)	1	
	Middle	4.10 (1.50)	1.11 (0.79-1.57)	.55	4.13 (1.51)	1.22 (0.86-1.73)	.27
	Upper	4.36 (1.49)	1.62 (0.60-4.34)	.34	4.38 (1.64)	1.72 (0.60-4.94)	.32
**Education level**							
	No schooling completed	4.00 (1.41)	1		4.00 (0.93)	1	
	Some primary school	3.88 (1.94)	1.34 (0.21-8.62)	.76	3.47 (1.94)	0.42 (0.07-2.47)	.33
	Completed primary school	4.26 (1.66)	2.99 (0.55-16.37)	.21	3.76 (1.78)	0.83 (0.17-4.17)	.82
	Some secondary school	4.08 (1.62)	2.36 (0.46-12.28)	.31	4.38 (1.67)	1.86 (0.39-8.89)	.44
	Completed secondary school	3.85 (1.60)	1.58 (0.32-7.80)	.57	3.89 (1.62)	0.85 (0.19-3.83)	.83
	Diploma, advanced diploma, associate degree or equivalent	4.02 (1.36)	2.11 (0.41-10.82)	.37	3.86 (1.50)	0.87 (0.18-4.07)	.86
	Bachelor’s degree	4.12 (1.38)	2.16 (0.42-11.00)	.35	4.12 (1.24)	0.94 (0.20-4.38)	.94
	Master’s degree	4.29 (1.52)	2.65 (0.50-13.89)	.25	4.26 (1.68)	1.03 (0.21-5.01)	.97
	Doctoral degree	3.72 (1.52)	1.09 (0.17-6.95)	.93	4.42 (1.39)	1.15 (0.20-6.66)	.88
	Other	7.00 (0)	N/A	N/A	5.00 (0)	2.15 (0.08-59.35)	.65
**Occupation**							
	Service	3.68 (1.43)	1		3.79 (1.53)	1	
	Sales	3.21 (1.28)	0.65 (0.27-1.57)	.34	3.30 (1.45)	0.74 (0.31-1.81)	.51
	Catering	4.30 (1.35)	2.31 (0.67-7.96)	.19	4.50 (1.20)	2.68 (0.79-9.09)	.11
	Finance	4.30 (1.54)	1.90 (0.90-4.01)	.09	4.28 (1.28)	2.03 (0.97-4.26)	.06
	Engineering	4.30 (1.24)	1.88 (0.96-3.68)	.07	4.08 (1.44)	1.38 (0.70-2.70)	.35
	Art	3.75 (0.83)	0.99 (0.19-5.08)	.99	4.75 (0.83)	3.40 (0.64-18.10)	.15
	Education/culture/academia	4.27 (1.53)	2.31 (1.16-4.59)	.02	4.42 (1.43)	2.18 (1.10-4.34)	.03
	Administration/professional	3.71 (1.47)	1.00 (0.46-2.14)	.99	4.17 (1.48)	1.58 (0.74-3.40)	.24
	Office/white-collar worker	4.12 (1.30)	1.96 (0.88-4.36)	>.99	3.89 (1.28)	1.43 (0.66-3.11)	.37
	Disciplinary forces	4.86 (1.46)	5.07 (1.25-20.62)	.02	4.29 (1.48)	1.80 (0.45-7.23)	.41
	Student	3.88 (1.23)	1.37 (0.68-2.72)	.38	3.98 (1.31)	1.54 (0.76-3.10)	.23
	Housewife/househusband	4.12 (1.65)	1.91 (0.90-4.05)	.09	3.95 (1.84)	1.23 (0.57-2.64)	.60
	Unemployed/awaiting job assignment	4.00 (1.66)	1.58 (0.60-4.21)	.36	3.56 (1.62)	0.81 (0.30-2.19)	.68
	Retiree	4.11 (1.77)	1.28 (0.65-2.52)	.48	3.95 (1.77)	1.11 (0.57-2.19)	.76
	Other	4.38 (1.47)	2.21 (0.94-5.17)	.07	4.46 (1.35)	2.50 (1.07-5.82)	.03

## Discussion

### Mobile Device Use

The results of this study contribute to the evidence for the high penetration of mobile devices, with almost every participant having a mobile device of some kind. Mobile phone users comprised over 90% of the participants. This is significantly higher than the mobile phone user rates reported in previous studies [8,9], which could be due to the proliferation of mobile phones in Asia in recent years [16].

### Prevalence and Demographic Correlates of Health App Use

In this study, approximately one-third of the mobile device users reported using health apps. The most prevalent types of health apps were those that can help individuals obtain more health information, track their vital signs, or receive health and medical reminders. The popularity of these apps could be related to the fact that individuals are now increasingly interested in managing their diets and lifestyles to stay healthy [17]. Moreover, we suggest that the popularity of health apps is related to their usability because apps that are easier to use might increase users’ self-efficacy and willingness to use them [17].

We found that people in higher self-rated social classes were more likely to use health apps, especially apps offering healthy living information and recovery and rehabilitation information, and apps measuring/recording vital signs. One reason for this could be that individuals in higher self-rated social classes are more health conscious, as research has shown that individuals in higher social classes are more likely to think about how to stay healthy [18]. Another reason could be that individuals in higher social classes find it easier to pay for apps or mobile technology (eg, wearable devices) that is often used with health apps [9,19].

We also found that women were more likely to use health apps than men, particularly health and medical reminder apps. This might be because women care more about healthy living than men; for instance, women attach greater importance to healthy eating than men [20]. There is also evidence that women better adhere to public health recommendations for exercise, tobacco and alcohol consumption, and healthy diets [21]. In addition to the gender differences in attitudes toward healthy living, motherhood might be one reason that women used health apps more than men because women have more need for apps related to pregnancy, postnatal recovery, baby care, etc.

It was also noted that participants who had obtained medium-level education (ie, completed secondary school or obtained a diploma, advanced diploma, or equivalent degree) were more likely to use diagnosis assistance apps than participants with either lower or higher education levels. As indicated by previous research, individuals whose education levels were lower than secondary schooling tended to have lower health literacy, which may be why they were less likely to use diagnosis assistance apps [22]. Individuals who had obtained bachelor’s degrees or higher might have more trust in health care professionals, and might thus tend to visit physicians for diagnoses instead of conducting self-diagnosis using health apps [23].

Retirees and housewives/househusbands were less likely to use certain types of apps (eg, diagnosis assistance apps, and health and medical reminder apps). This could be because they live life at a slower pace and are less likely to be occupied with work, so have less need for reminder apps or apps assisting with immediate self-diagnosis. We also found that participants working in the art industry were more likely to use recovery and rehabilitation information apps, but the underlying reason for this is less clear.

### Perceived Impacts of Health App Use on Health Knowledge and Health Condition Management

Research has shown that health apps have the potential to promote healthy behaviors, facilitate health management, and improve health outcomes [10-13]. However, only slightly more than one-third of the participants in our study held positive opinions about the impacts of using health apps. The reason for this could be that most of the participants had not used any health apps before and were not aware of their potential benefits.

Participants with occupations related to health or education (eg, health care professionals, education/culture/academic professionals) or those whose occupations required a high level of physical fitness (eg, sports players, disciplinary forces) were more likely to perceive health apps as useful. This might be because the participants in these occupations had higher health literacy and better understood how to use health apps to improve their health outcomes.

### Implications for Future Research

To better understand the reasons for health app use disparities among different demographic groups, knowledge about why or why not individuals use health apps is needed. This has not been thoroughly studied. We suggest that more qualitative research should be conducted to explore the facilitators of and barriers to health app use. Moreover, most studies examining the impacts of health apps focused on their impacts on health outcomes, whereas their impacts on resource utilization were less studied. Health apps have the potential to save time and money for their users, reduce hospitalization, cut costs, and reduce necessary human resources in health care, but these effects need to be further validated. Thus, we suggest that more research be done to examine whether using health apps can improve resource utilization.

### Implications for Policy

With so many health apps now available, people might find it difficult to identify quality health apps that match their needs and are trustworthy. The European Commission and the US Food and Drug Administration have offered guidelines to regulate health apps to assure their safety and effectiveness [24,25]; however, these guidelines only apply to a small number of health apps [26]. In addition to controlling the quality of health apps, guidelines or recommendations that help people choose appropriate health apps for their needs are also important. Governmental health agencies or other influential health organizations could consider developing standardized health app evaluation criteria and a decision-making framework to help people evaluate health apps and choose the apps they need.

### Implications for Practice

Despite the fact that health apps can be convenient and useful, we found that most people were not aware of their benefits. We suggest that effort be made to promote health apps, especially to demographic groups that are less likely to use health apps (eg, males, individuals from lower social classes), to facilitate health management and improve individual health outcomes.

Usability is a prerequisite for widespread use of health apps. However, research has found significant usability barriers for health apps that are currently available and suggested that their usability needs to be improved [27]. Moreover, we suggest that app developers consider the needs of individuals with low health and technology literacy so that even people with little knowledge about health or mobile technology can easily learn to use health apps. This might not only promote the use of health apps, but also help ameliorate health app use disparities.

### Conclusions

Despite the prevalence of mobile devices, many people have never used any mobile health apps. In fact, many of them are unaware of the potential benefits of using health apps. Effort should be made to promote health apps, especially to demographic groups that are less likely to use health apps. Health organizations and agencies could help individuals identify quality health apps that meet their needs by developing standardized health app evaluation criteria and a decision-making framework for choosing health apps. App developers should improve the usability of health apps so that even people with little knowledge about health and mobile technology can easily learn to use them.

## Appendix

Multimedia Appendix 1 Study questionnaire.

Multimedia Appendix 2 Prevalence, extent, and demographic correlates of health app use (N=612).

## Abbreviations

OR: odds ratio
