# Supplementary material for: Prevalence, Demographic Correlates, and Perceived Impacts of Mobile Health App Use Amongst Chinese Adults: Cross-Sectional Survey Study
Source: JMIR Mhealth Uhealth. 2018 Apr 26;6(4):e103. doi: 10.2196/mhealth.9002 (PMC5945985; doi:10.2196/mhealth.9002)
Supplement: Multimedia Appendix 1 [file mhealth_v6i4e103_app1.pdf]

Appendix Table A. Study questionnaire

Demographic

Gender	☐ Male ☐ Female
Age	____ years
Social class (circle the number that best describes your social class):	Poor Middle class Rich<br>1 2 3 4 5 6 7 8 9
Your education level:<br><br>☐ No schooling completed<br>☐ Some primary school<br>☐ Completed primary school<br>☐ Some secondary school<br>☐ Completed secondary school<br>☐ Diploma, advanced diploma, associate degree or equivalent<br>☐ Bachelor’s degree<br>☐ Master’s degree<br>☐ Doctoral degree<br>☐ Other (please specify): _____________	
Your occupation group:	☐ Service<br>☐ Sales<br>☐ Catering<br>☐ Finance<br>☐ Engineering<br>☐ Art<br>☐ Education/culture/academia<br>☐ Administration/professional<br>☐ Office/white-collar worker<br>☐ Disciplinary forces (e.g., police force, immigration department, customs and excise department, fire services department, correctional services department)<br>☐ Student<br>☐ Housewife/house-husband<br>☐ Unemployed/awaiting job assignment<br>☐ Retiree<br>☐ Other (please specify): _____________

Use of mobile devices

Do you use any of the following mobile devices?	On average, how much time do you spend each day using the device?
☐ Smartphone	___ hrs ___ mins
☐ Feature phone	___ hrs ___ mins
☐ Tablet computer	___ hrs ___ mins
☐ Other (please specify): _____________	___ hrs ___ mins
☐ I don’t use any mobile devices	

Internet access on mobile devices

Do you have Internet access on your mobile device?	☐ Yes ☐ No

Average length of time of each occasion of use

Use of health applications

☐ Healthy living information (e.g., diet, nutrition, exercise)Frequency of use:<br>1 – Several times a day<br>2 – Once or twice a day<br>3 – Several times a week<br>4 – Once or twice a week<br>5 – Several times a month	1 2 3 4 5 6 7	___ hrs ___ mins

6 – Once or twice a month<br>7 – Never<br>Do you have any of the types of medical/health applications listed below installed on your mobile device? How frequently do you use them?		
☐ Recovery and rehabilitation information (e.g., physical therapy instruction)	1 2 3 4 5 6 7	___ hrs ___ mins
☐ Diagnosis assistance (e.g., symptom checking)	1 2 3 4 5 6 7	___ hrs ___ mins
☐ Telehealth (e.g., remote monitoring)	1 2 3 4 5 6 7	___ hrs ___ mins
☐ Health and medical reminders (e.g., period tracking, medication reminders)	1 2 3 4 5 6 7	___ hrs ___ mins
☐ Emergency services (e.g., fall detection)	1 2 3 4 5 6 7	___ hrs ___ mins
☐ Measuring/recording vital signs (e.g., body temperature, heart rate, blood pressure)	1 2 3 4 5 6 7	___ hrs ___ mins
☐ Other (please specify): ______________	1 2 3 4 5 6 7	___ hrs ___ mins
☐ I don't have any medical/health applications installed on my mobile device		
**Perceived impacts of using health apps**		
Using mobile medical/health applications can increase your knowledge about your own health conditions.		
Using mobile medical/ health applications can improve the effectiveness of the management of your health conditions.***Very Strongly Disagree*** ***1 2 3 4 5 6 7*** ***Very Strongly Agree***	Very Strongly Disagree 1 2 3 4 5 6 7 Very Strongly Agree	
