# Supplementary material for: Prevalence, Demographic Correlates, and Perceived Impacts of Mobile Health App Use Amongst Chinese Adults: Cross-Sectional Survey Study
Source: JMIR Mhealth Uhealth. 2018 Apr 26;6(4):e103. doi: 10.2196/mhealth.9002 (PMC5945985; doi:10.2196/mhealth.9002)
Supplement: Multimedia Appendix 2 [file mhealth_v6i4e103_app2.pdf]

Prevalence, extent, and demographic correlates of health app use (N=612).

	Healthy living information			
	n (%)	Usage[a]	OR (95% CI)	*P*
**Total**	197 (32.2)	44.60		
**Gender**				
Male	93 (28.6)	33.06	1	
Female	103 (33.6)	56.18	1.37 (0.92, 2.06)	.12
**Age**				
18-29	51 (32.7)	29.70	1	
30-44	59 (37.3)	45.59	1.06 (0.61, 1.86)	.83
45-59	51 (32.7)	70.36	0.98 (0.54, 1.81)	.96
60 or older	32 (20.3)	25.11	0.79 (0.35, 1.78)	.57
**Self-rated social class**				
Lower	54 (25.7)	32.73	1	
Middle	134 (33.3)	50.60	1.55 (1.01, 2.40)	.046
Upper	7 (50.0)	20.17	2.84 (0.88, 9.08)	.08
**Education level**				
No schooling completed	0 (0)	N/A	1	
Some primary school	2 (11.1)	30.00	0.27 (0.04, 1.85)	.18
Completed primary school	9 (24.3)	96.60	0.63 (0.16, 2.55)	.52
Some secondary school	20 (37.0)	30.78	0.95 (0.27, 3.38)	.94
Completed secondary school	46 (26.3)	32.62	0.53 (0.17, 1.65)	.27
Diploma, advanced diploma, associate degree or equivalent	25 (27.8)	20.25	0.50 (0.16, 1.59)	.24
Bachelor’s degree	56 (36.4)	41.38	0.55 (0.18, 1.62)	.28
Master’s degree	31 (40.3)	87.29	0.71 (0.24, 2.12)	.53
Doctoral degree	8 (42.1)	17.29	1	
Other	0 (0)	N/A	1	
**Occupation**				
Service	30 (36.1)	34.80	1	
Sales	7 (29.2)	36.67	0.71 (0.25, 2.00)	.51
Catering	1 (7.7)	NR[b]	0.19 (0.02, 1.60)	.13
Finance	17 (41.5)	24.09	1.12 (0.47, 2.69)	.79
Engineering	16 (32.7)	26.21	0.77 (0.33, 1.78)	.54
Art	1 (25.0)	NR[b]	0.57 (0.05, 6.17)	.64
Education/culture/academia	20 (31.3)	130.59	0.59 (0.26, 1.36)	.22
Administration/professional	21 (60.0)	36.39	2.35 (0.95, 5.82)	.07
Office/white-collar worker	12 (34.3)	27.80	0.83 (0.32, 2.15)	.70
Disciplinary forces	3 (42.9)	30.00	1.27 (0.25, 6.35)	.77
Student	19 (29.2)	41.94	0.61 (0.26, 1.45)	.27
Housewife/house-husband	12 (26.7)	30.60	0.60 (0.25, 1.43)	.25
Unemployed/awaiting job assignment	5 (31.3)	20.00	0.86 (0.26, 2.84)	.81
Retiree	22 (18.0)	30.31	0.50 (0.22, 1.16)	.11
Other	9 (37.5)	80.00	1.12 (0.41, 3.09)	.83

(Table continued)

	Recovery and rehabilitation information			
	n (%)	Usage[a]	OR (95% CI)	*P*
**Total**	42 (6.9)	32.30		
**Gender**				
Male	26 (8.0)	35.23	1	
Female	16 (5.2)	26.36	0.72 (0.34, 1.55)	.40
**Age**				
18-29	4 (2.6)	50.00	1	
30-44	7 (4.4)	15.6	1.43 (0.33, 6.28)	.63

45-59	15 (9.6)	40.00	3.62 (0.87, 15.11)	.08
60 or older	15 (9.5)	29.00	4.10 (0.85, 19.78)	.08
**Self-rated social class**				
Lower	8 (3.8)	22.16	1	
Middle	31 (7.7)	36.08	2.98 (1.14, 7.80)	.03
Upper	2 (14.3)	3.00	15.01 (2.03, 110.78)	.01
**Education level**				
No schooling completed	0 (0)	N/A	1	
Some primary school	1 (5.6)	30.00	1.48 (0.10, 21.53)	.78
Completed primary school	2 (5.4)	14.99	1.91 (0.23, 15.88)	.55
Some secondary school	4 (7.4)	41.33	1.83 (0.27, 12.49)	.54
Completed secondary school	19 (10.9)	31.58	4.04 (0.92, 17.79)	.07
Diploma, advanced diploma, associate degree or equivalent	6 (6.7)	38.33	2.66 (0.50, 14.3)	.25
Bachelor’s degree	7 (4.5)	36.17	1.22 (0.27, 5.63)	.80
Master’s degree	3 (3.9)	18.33	1	
Doctoral degree	0 (0)	N/A	1	
Other	0 (0)	N/A	1	
**Occupation**				
Service	9 (10.8)	29.50	1	
Sales	5 (20.8)	18.75	3.34 (0.79, 14.18)	.10
Catering	0 (0)	N/A	1	
Finance	2 (4.9)	30.00	0.95 (0.16, 5.82)	.96
Engineering	1 (2.0)	20.00	1	
Art	1 (25.0)	NR[b]	20.37 (1.29, 320.51)	.03
Education/culture/academia	1 (1.6)	15.00	0.25 (0.03, 2.26)	.22
Administration/professional	4 (11.4)	32.50	1.52 (0.35, 6.70)	.58
Office/white-collar worker	0 (0)	N/A	1	
Disciplinary forces	0 (0)	N/A	1	
Student	1 (1.5)	120.00	0.35 (0.03, 4.30)	.41
Housewife/house-husband	2 (4.4)	20.00	0.40 (0.07, 2.24)	.30
Unemployed/awaiting job assignment	3 (18.8)	15.00	2.82 (0.57, 13.94)	.20
Retiree	9 (7.4)	40.33	0.40 (0.11, 1.40)	.15
Other	3 (12.5)	5.00	1.56 (0.32, 7.57)	.58

(Table continued)

	Diagnosis assistance			
	n (%)	Usage[a]	OR (95% CI)	*P*
**Total**	28 (4.6)	26.20		
**Gender**				
Male	15 (4.6)	30.50	1	
Female	12 (3.9)	21.87	1.29 (0.46, 3.63)	.63
**Age**				
18-29	5 (3.2)	11.65	1	
30-44	5 (3.2)	19.75	0.90 (0.19, 4.24)	.90
45-59	9 (5.8)	41.43	1.12 (0.25, 5.08)	.88
60 or older	8 (5.1)	24.50	2.38 (0.46, 12.29)	.30
**Self-rated social class**				
Lower	8 (3.8)	16.49	1	
Middle	18 (4.5)	30.00	2.74 (0.88, 8.51)	.08
Upper	1 (7.1)	2.00	111.09 (4.31, 2828.89)	.004
**Education level**				
No schooling completed	0 (0)	N/A	1	
Some primary school	0 (0)	N/A	1	
Completed primary school	1 (2.7)	10.00	12.04 (0.43, 334.09)	.14

Some secondary school	3 (5.6)	37.50	12.71 (0.66, 246.02)	.09
Completed secondary school	16 (9.1)	23.27	35.68 (2.85, 447.02)	.01
Diploma, advanced diploma, associate degree or equivalent	5 (5.6)	20.00	15.55 (1.07, 225.77)	.04
Bachelor’s degree	3 (1.9)	43.32	1	
Master’s degree	0 (0)	N/A	1	
Doctoral degree	0 (0)	N/A	1	
Other	0 (0)	N/A	1	
**Occupation**				
Service	8 (9.6)	29.80	1	
Sales	5 (20.8)	30.00	3.11 (0.66, 14.57)	.15
Catering	0 (0)	N/A	1	
Finance	1 (2.4)	30.00	0.92 (0.08, 10.41)	.95
Engineering	2 (4.1)	7.50	0.48 (0.05, 4.45)	.52
Art	0 (0)	N/A	1	
Education/culture/academia	0 (0)	N/A	1	
Administration/professionals	4 (11.4)	23.75	2.48 (0.49, 12.53)	.27
Office/white-collar worker	0 (0)	N/A	1	
Disciplinary forces	0 (0)	N/A	1	
Student	1 (1.5)	9.96	1	
Housewife/house-husband	1 (2.2)	10.00	0.16 (0.02, 1.54)	.11
Unemployed/awaiting job assignment	1 (6.3)	NR[b]	1.05 (0.11, 10.39)	.97
Retiree	2 (1.6)	31.00	0.06 (0.01, 0.43)	.01
Other	2 (8.3)	NR[b]	1.15 (0.17, 7.96)	.88

(Table continued)

	Telehealth			
	n (%)	Usage[a]	OR (95% CI)	*P*
**Total**	11 (1.8)	44.57		
**Gender**				
Male	7 (2.2)	25.00	1	
Female	4 (1.3)	30.00	1.62 (0.31, 8.45)	.57
**Age**				
18-29	0 (0)	N/A	1	
30-44	0 (0)	N/A	1	
45-59	5 (3.2)	45.00	0.43 (0.07, 2.86)	.39
60 or older	5 (3.2)	21.67	1	
**Self-rated social class**				
Lower	3 (1.4)	NR[b]	1	
Middle	6 (1.5)	30.00	0.71 (0.09, 5.69)	.75
Upper	1 (7.1)	5.00	26.93 (0.77, 937.96)	.07
**Education level**				
No schooling completed	0 (0)	N/A	1	
Some primary school	0 (0)	N/A	1	
Completed primary school	0 (0)	N/A	1	
Some secondary school	1 (1.9)	60.00	1	
Completed secondary school	7 (4.0)	17.50	2.98 (0.18, 50.76)	.45
Diploma, advanced diploma, associate degree or equivalent	2 (2.2)	NR[b]	1.75 (0.05, 57.87)	.76
Bachelor’s degree	1 (0.6)	60.00	1	
Master’s degree	0 (0)	N/A	1	
Doctoral degree	0 (0)	N/A	1	
Other	0 (0)	N/A	1	
**Occupation**				
Service	4 (4.8)	60.00	1	
Sales	1 (4.2)	NR[b]	3.21 (0.17, 59.22)	.43

Catering	0 (0)	N/A	1	
Finance	0 (0)	N/A	1	
Engineering	1 (2.0)	NR[b]	1	
Art	0 (0)	N/A	1	
Education/culture/academia	0 (0)	N/A	1	
Administration/professional	1 (2.9)	30.00	1.3 (0.09, 19.79)	.85
Office/white-collar worker	0 (0)	N/A	1	
Disciplinary forces	0 (0)	N/A	1	
Student	0 (0)	N/A	1	
Housewife/house-husbands	0 (0)	N/A	1	
Unemployed/awaiting job assignment	0 (0)	N/A	1	
Retiree	2 (1.6)	32.50	0.14 (0.01, 1.54)	.11
Other	2 (8.3)	NR[b]	4.69 (0.38, 57.33)	.23

(Table continued)

	Health and medical reminders			
	n (%)	Usage[a]	OR (95% CI)	*P*
**Total**	64 (10.5)	32.27		
**Gender**				
Male	24 (7.4)	62.71	1	
Female	39 (12.7)	8.70	2.44 (1.31, 4.52)	.01
**Age**				
18-29	23 (14.7)	34.46	1	
30-44	18 (11.4)	5.92	0.79 (0.35, 1.78)	.57
45-59	12 (7.7)	87.14	0.63 (0.25, 1.60)	.33
60 or older	10 (6.3)	17.75	1.16 (0.35, 3.86)	.80
**Self-rated social class**				
Lower	19 (9.0)	5.40	1	
Middle	41 (10.2)	40.75	1.42 (0.70, 2.88)	.33
Upper	3 (21.4)	8.00	2.99 (0.68, 13.20)	.15
**Education level**				
No schooling completed	0 (0)	N/A	1	
Some primary school	0 (0)	N/A	1	
Completed primary school	1 (2.7)	10.00	0.36 (0.02, 7.67)	.51
Some secondary school	2 (3.7)	32.50	0.22 (0.01, 4.53)	.32
Completed secondary school	18 (10.3)	32.57	1.56 (0.16, 15.26)	.70
Diploma, advanced diploma, associate degree or equivalent	10 (11.1)	81.89	1.86 (0.19, 18.51)	.60
Bachelor's degree	20 (13.0)	10.46	1.80 (0.20, 16.65)	.60
Master's degree	12 (15.6)	11.25	2.47 (0.27, 22.63)	.42
Doctoral degree	1 (5.3)	5.00	1	
Other	0 (0)	N/A	1	
**Occupation**				
Service	13 (15.7)	12.00	1	
Sales	3 (12.5)	NR[b]	0.46 (0.11, 1.92)	.29
Catering	0 (0)	N/A	1	
Finance	4 (9.8)	5.00	0.29 (0.07, 1.12)	.07
Engineering	3 (6.1)	165.00	0.21 (0.04, 1.10)	.06
Art	2 (50.0)	3.00	3.10 (0.34, 28.70)	.32
Education/culture/academia	6 (9.4)	13.75	0.31 (0.09, 1.04)	.06
Administration/professional	8 (22.9)	13.33	0.75 (0.24, 2.36)	.63
Office/white-collar worker	3 (8.6)	7.33	0.28 (0.07, 1.16)	.08
Disciplinary forces	0 (0)	N/A	1	
Student	9 (13.8)	54.29	0.33 (0.10, 1.10)	.07
Housewife/house-husband	2 (4.4)	10.00	0.16 (0.03, 0.82)	.03

Unemployed/awaiting job assignment	0 (0)	N/A	1	
Retiree	6 (4.9)	24.40	0.23 (0.06, 0.88)	.03
Other	5 (20.8)	10.00	0.97 (0.26, 3.56)	.97

(Table continued)

	Emergency services			
	n (%)	Usage[a]	OR (95% CI)	*P*
**Total**	16 (2.6)	26.24		
**Gender**				
Male	10 (3.1)	15.56	1	
Female	6 (2.0)	6.67	0.89 (0.25, 3.14)	.85
**Age**				
18-29	1 (0.6)	NR[b]	1	
30-44	4 (2.5)	2.50	3.03 (0.32, 29.03)	.34
45-59	4 (2.6)	40.00	1.88 (0.17, 20.85)	.61
60 or older	6 (3.8)	12.50	8.07 (0.66, 98.10)	.10
**Self-rated social class**				
Lower	4 (1.9)	NR[b]	1	
Middle	11 (2.7)	10.00	2.06 (0.48, 8.82)	.33
Upper	0 (0)	N/A	1	
**Education level**				
No schooling completed	0 (0)	N/A	1	
Some primary school	0 (0)	N/A	1	
Completed primary school	0 (0)	N/A	1	
Some secondary school	4 (7.4)	20.00	3.75 (0.25, 56.91)	.34
Completed secondary school	5 (2.9)	10.00	2.02 (0.17, 23.80)	.58
Diploma, advanced dip, associate degree or equivalent	3 (3.3)	NR[b]	1.92 (0.13, 27.37)	.63
Bachelor's degree	3 (1.9)	25.00	2.23 (0.20, 24.36)	.51
Master's degree	1 (1.3)	5.00	1	
Doctoral degree	0 (0)	N/A	1	
Others	0 (0)	N/A	1	
**Occupations**				
Service	5 (6.0)	30.00	1	
Sales	1 (4.2)	NR[b]	0.73 (0.06, 8.16)	.80
Catering	0 (0)	N/A	1	
Finance	1 (2.4)	NR[b]	0.51 (0.04, 6.77)	.61
Engineering	2 (4.1)	NR[b]	0.47 (0.04, 5.17)	.54
Art	0 (0)	N/A	1	
Education/culture/academia	0 (0)	N/A	1	
Administration/professional	3 (8.6)	8.33	2.05 (0.30, 14.06)	.46
Office/white-collar worker	0 (0)	N/A	1	
Disciplinary forces	0 (0)	N/A	1	
Student	0 (0)	N/A	1	
Housewife/house-husband	1 (2.2)	NR[b]	0.51 (0.04, 5.78)	.58
Unemployed/awaiting job assignment	0 (0)	N/A	1	
Retiree	3 (2.5)	25.00	0.21 (0.03, 1.34)	.10
Other	0 (0)	N/A	1	

(Table 4 continued)

	Measuring/recording vital signs			
	n (%)	Usage[a]	OR (95% CI)	*P*
**Total**	80 (13.1)	25.83		
**Gender**				
Male	52 (16.0)	23.15	1	
Female	28 (9.1)	32.55	0.71 (0.40, 1.26)	.24

**Age**				
18-29	14 (9.0)	17.12	1	
30-44	18 (11.4)	46.33	1.08 (0.45, 2.55)	.87
45-59	26 (16.7)	22.95	2.01 (0.84, 4.83)	.12
60 or older	19 (12.0)	17.81	2.28 (0.76, 6.87)	.14
**Self-rated social class**				
Lower	15 (7.1)	59.28	1	
Middle	57 (14.2)	23.14	2.31 (1.16, 4.60)	.02
Upper	5 (35.7)	3.20	8.32 (2.16, 32.05)	.002
**Education level**				
No schooling completed	0 (0)	N/A	1	
Some primary school	1 (5.6)	3.00	0.47 (0.03, 6.62)	.58
Completed primary school	1 (2.7)	3.00	0.19 (0.01, 2.49)	.21
Some secondary school	4 (7.4)	45.00	0.38 (0.05, 2.66)	.33
Completed secondary school	24 (13.7)	17.18	0.94 (0.19, 4.52)	.93
Diploma, advanced diploma, associate degree or equivalent	13 (14.4)	42.44	1.03 (0.21, 5.05)	.97
Bachelor’s degree	21 (13.6)	38.50	0.79 (0.18, 3.60)	.77
Master’s degree	13 (16.9)	16.36	1.04 (0.23, 4.70)	.96
Doctoral degree	3 (15.8)	10.67	1	
Other	0 (0)	N/A	1	
**Occupation**				
Service	12 (14.5)	55.62	1	
Sales	5 (20.8)	31.67	1.48 (0.41, 5.28)	.55
Catering	0 (0)	N/A	1	
Finance	6 (14.6)	15.00	0.93 (0.27, 3.15)	.91
Engineering	9 (18.4)	38.00	0.73 (0.24, 2.24)	.59
Art	0 (0)	N/A	1	
Education/culture/academia	9 (14.1)	12.00	0.68 (0.22, 2.09)	.50
Administration/professional	6 (17.1)	21.17	0.94 (0.28, 3.13)	.91
Office/white-collar worker	4 (11.4)	47.50	0.69 (0.16, 2.94)	.62
Disciplinary forces	2 (28.6)	10.00	1.85 (0.29, 11.54)	.51
Student	5 (7.7)	7.50	0.53 (0.14, 2.04)	.35
Housewife/house-husband	1 (2.2)	19.98	0.15 (0.02, 1.25)	.08
Unemployed/awaiting job assignment	2 (12.5)	2.00	0.77 (0.14, 4.12)	.76
Retiree	14 (11.5)	20.00	0.42 (0.14, 1.24)	.12
Other	4 (16.7)	10.00	1.04 (0.27, 4.04)	.95

(Table continued)

	Other health apps			
	n (%)	Usage[a]	OR (95% CI)	*P*
**Total**	19 (3.1)	31.00		
**Gender**				
Male	11 (3.4)	36.50	1	
Female	8 (2.6)	148.40	0.72 (0.25, 2.12)	.56
**Age**				
18-29	4 (2.6)	330.00	1	
30-44	2 (1.3)	2.00	0.44 (0.07, 2.90)	.40
45-59	11 (7.1)	34.67	2.88 (0.62, 13.26)	.18
60 or older	2 (1.3)	60.00	0.67 (0.07, 6.36)	.73
**Self-rated social class**				
Lower	6 (2.9)	23.00	1	
Middle	12 (3.0)	110.25	1.21 (0.39, 3.79)	.75
Upper	0 (0)	N/A	1	
**Education level**				
No schooling completed	0 (0)	N/A	1	

Some primary school	0 (0)	N/A	1	
Completed primary school	0 (0)	N/A	1	
Some secondary school	3 (5.6)	35.00	1.49 (0.10, 22.41)	.77
Completed secondary school	7 (4.0)	28.00	1.52 (0.15, 15.19)	.72
Diploma, advanced diploma, associate degree or equivalent	4 (4.4)	43.33	2.52 (0.24, 26.42)	.44
Bachelor's degree	4 (2.6)	240.00	1.55 (0.15, 15.46)	.71
Master's degree	1 (1.3)	2.00	1	
Doctoral degree	0 (0)	N/A	1	
Other	0 (0)	N/A	1	
**Occupation**				
Service	7 (8.4)	37.5	1	
Sales	0 (0)	N/A	1	
Catering	1 (7.7)	60.00	1.32 (0.13, 13.72)	.82
Finance	1 (2.4)	600.00	0.54 (0.05, 6.25)	.63
Engineering	0 (0)	N/A	1	
Art	0 (0)	N/A	1	
Education/culture/academia	0 (0)	N/A	1	
Administration/professional	1 (2.9)	2.00	0.47 (0.05, 4.87)	.53
Office/white-collar worker	2 (5.7)	60.00	1.39 (0.21, 9.30)	.73
Disciplinary forces	1 (14.3)	30.00	1.44 (0.13, 15.95)	.77
Student	1 (1.5)	NR[b]	0.32 (0.02, 4.11)	.38
Housewife/house-husband	1 (2.2)	2.00	0.39 (0.04, 3.91)	.42
Unemployed/awaiting job assignment	0 (0)	N/A	1	
Retiree	2 (1.6)	35.00	0.38 (0.05, 2.81)	.34
Other	2 (8.3)	60.00	2.21 (0.34, 14.35)	.41

[a] Mean length of time spent on each occasion of use, measured in minutes

[b] Participants did not respond to the question
